# Measuring health care experiences that matter to Indigenous people in Australia with cancer: identifying critical gaps in existing tools

**DOI:** 10.1186/s12939-021-01433-2

**Published:** 2021-04-12

**Authors:** Monica Green, Joan Cunningham, Kate Anderson, Kalinda Griffiths, Gail Garvey

**Affiliations:** 1 Wellbeing and Preventable Chronic Diseases Division, Menzies School of Health Research, Charles Darwin University, Level 10, East Tower, 410 Ann Street, Brisbane, QLD 4000 Australia; 2 Centre for Big Data Research in Health, University of New South Wales, Sydney, Australia

**Keywords:** Indigenous, Aboriginal, Australia, Cancer, Patient experience, Measurement, Person-centred care

## Abstract

**Background:**

Measurement of patients’ healthcare experiences is increasingly used as an indicator of quality of care, but there are concerns that existing measures omit information that is meaningful to patients and that results may not be used systematically to inform service improvement. Further, current approaches may be inadequate for some population groups, such as Indigenous people in Australia, whose healthcare experience is impacted by the context of colonisation and discordance between Indigenous understandings of health and the Western biomedical health system. This study aimed to assess the extent to which existing patient experience measures used in Australia collect information about critical aspects of cancer care, as previously identified by Indigenous people affected by cancer and their health care providers.

**Methods:**

A two-stage process was used to examine the adequacy of existing patient experience measures for Indigenous people in Australia: (1) relevant tools and measures were identified and assessed, and four measures selected as suitable comparators; (2) comparators were examined in detail and mapped against topics identified in earlier research as important to Indigenous people with cancer. Gaps in topic coverage in comparators were identified.

**Results:**

No comparators completely captured the critical aspects of cancer care identified by Indigenous people affected by cancer and their health care providers. The number of topics ‘partially’ captured by the four comparators ranged from 4 to 7 out of 9. While most topics were partially covered, the lack of questions around culture and cultural safety was notable.

**Conclusions:**

Existing tools are likely to miss key aspects of Indigenous peoples’ experiences of cancer care in Australia. Failure to adequately assess care experiences related to cultural safety may compromise efforts to improve health outcomes. Addressing gaps requires development of experience measures that are strengths-based, reflect an Indigenous worldview and measure aspects of experience relevant to Indigenous people.

**Supplementary Information:**

The online version contains supplementary material available at 10.1186/s12939-021-01433-2.

## Background

Patient-reported experiences are routinely used as a key indicator of the quality of health care, along with patient-reported outcome measures, safety measures and clinical outcomes [1–3]. However, there are concerns that many mechanisms used to collect patient experience data produce large amounts of superficial data, while missing deeper, more nuanced information that is meaningful to the patient [4, 5], and that insufficient attention is paid to whether the information is used to inform health care provision [6–8]. In addition, gaps have been identified in understanding the experiences of underserved groups, whose interactions with health services may be markedly different to general patient populations [9]. In Australia, patient experience surveys have been reported as unsuitable for people who speak little or no English [10] and lacking in the ability to capture nuance in the experiences of some patient groups [4].

Recent efforts have been made to consider the adequacy of existing measures for Aboriginal and Torres Strait Islander (hereafter respectfully referred to as Indigenous) people in Australia. Deeper exploration is warranted, as significant systemic and cultural differences between the experiences of Indigenous and non-Indigenous patients have been reported [11, 12]. Indigenous people have experienced long-term systemic discrimination and racism, including within the health system, and a disproportionately high burden of disease [13]. Cancer is a significant contributor to the disease burden, with lower five-year survival [13] and lower rates of treatment concordant with guidelines [14, 15] for Indigenous people compared with non-Indigenous people in Australia. Many challenges exist in the delivery of high quality cancer care in Australia’s health system, due to the fragmented nature of cancer services, the length and logistical demands of treatment and sensitivity around diagnosis [15, 16]. In addition, issues with accessibility, availability, appropriateness and quality of care persist, and these are heightened for Indigenous people living in regional or remote areas who often need to travel to tertiary centres in urban areas for treatment [15–17]. Focusing on the care experiences of Indigenous people may illuminate issues previously reported as problematic, such as trust and engagement in the health system, [15, 16] and facilitate understanding of patients’ perspectives in order to guide improvements in care and health outcomes [3, 9].

Inadequate cultural safety of health services is consistently acknowledged as an impediment to successful engagement with care for Indigenous people both in Australia and internationally [18, 19]. The term ‘cultural safety’ is often erroneously used interchangeably with related terms. We have been guided by the following definition of cultural safety [20]:

*Cultural safety is determined by Aboriginal and Torres Strait Islander individuals, families and communities.*

*Culturally safe practise is the ongoing critical reflection of health practitioner knowledge, skills, attitudes, practising behaviours and power differentials in delivering safe , accessible and responsive healthcare free of racism.*

Recent work has led to clearly articulated components of cultural safety in healthcare. Elvidge et al. [21] grouped the key characteristics of cultural safety into five domains: positive communication between patients and hospital staff; negative communication between patients and hospital staff; trust between patients and hospital staff; hospital environment; and support for Aboriginal families and culture. These findings are broadly consistent with other work, such as research on cultural safety in the context of cancer care coordination pathways for Aboriginal patients [22], a systematic review of culturally safe healthcare communication [23], and the qualitative research which forms the basis of the current study [16]. The recent release of recommended survey questions for assessing Indigenous inpatients’ care experiences (yet to be validated) builds on this work [24].

There are sound reasons for purposefully seeking the perspective of Indigenous people regarding their care, in a way that is culturally appropriate. The Western biomedical health system does not easily accommodate Indigenous understandings of health, which are broad and holistic [25]. Cultural aspects of life are central to Indigenous world views [26, 27] and may be prioritised above treatment outcomes [17], which contrasts with the disease-oriented approach of the biomedical system. Patient experience measurement instruments may not reflect the world views and priorities of Indigenous people [9, 16], as has been shown regarding supportive care needs [28], health related quality of life [29], wellbeing [30] and social and emotional wellbeing [31]. Although there may be some aspects of existing standard measures that are relevant to Indigenous people, their use for Indigenous people presumes common definitions, values, needs and perceptions of health; a presumption which is often inappropriate [28–32]. Many existing patient experience measures originate from work that sought to improve the delivery of patient-centred care (PCC), through principles established by Western organisations, such as the Picker Institute [33]. While PCC domains and the measures developed under these principles include indicators that reflect culture and connection, examination of whether these issues are adequately captured for Indigenous people is required.

Privileging the voices of Indigenous peoples is an important first step to understanding their experiences of care. In the first phase of this program of work, we conducted qualitative interviews with Indigenous people affected by cancer and health care providers, to identify aspects of cancer care that were critical in shaping the patient experience for Indigenous people with cancer [16]. The responses of the two participant groups were consistent and complementary, and have therefore been combined. Several key themes were identified as central: feeling safe in the system; the importance of Indigenous care providers; barriers to care, particularly if receiving treatment away from one’s own Country or traditional homelands (connection to Country reflects a spiritual, emotional and cultural relationship to land, central to Indigenous identity [27]); the role of family and friends; effective communication and education; the transition to primary care; carers’ wellbeing; and palliative care. These results raised questions about the adequacy of existing patient experience measures to elicit relevant information that would inform health service changes that are meaningful for Indigenous people.

The aim of this study was to assess the extent to which patient experience measures currently in use in Australia collect information about critical aspects of cancer care, as identified by Indigenous people affected by cancer and health care providers [16].

## Methods

### Ethics and governance

No ethics approval was required for this study, as no human participants were involved. The program of work was undertaken by a team including two highly experienced Indigenous researchers and three non-Indigenous researchers.

### Study design

A two-stage process (both of which are described below) was used to examine the adequacy of existing patient experience measures for Indigenous people in Australia. In Stage 1, four patient experience measures were selected as suitable comparators. In Stage 2, the aforementioned topics, reported as important care experiences by Indigenous people and health care providers [16], were used as a baseline for a mapping process to determine the extent to which the topics were covered by the selected comparators. Stage 2 included the identification of gaps in the comparators, with respect to their coverage of the baseline topics.

### Stage 1: identification of relevant patient experience comparators

A scoping review was conducted by MG in 2015 (and updated in 2019), to review patient experience measurement activity in Australia and the implications of this for Indigenous people with cancer in Australia (unpub). Databases searched included PubMed, Indigenous HealthInfoNet, Medline and CINAHL, covering English language, full-text, peer reviewed journal articles between January 2000 and May 2019. Searches of websites of key health (e.g. National and State/Territory Health Departments, Aboriginal Community Controlled Health Organisation [ACCHO] peak bodies) and cancer (e.g. Cancer Councils) organisations were also conducted. One of the outcomes of the review was the identification of existing patient experience surveys and indicator sets used in Australia. All surveys and indicator sets identified were developed for use in the general population.

Two authors (MG, JC) reviewed all the identified surveys and indicator sets and discussed and agreed on the comparators to be included in Stage 2. Selection of comparators was limited to those relating to adult (≥18 years old) experiences of care in the Australian health setting from 2012, with more recent surveys and indicators given more weight. Cancer-related comparators were of particular interest, as were those with direct relevance to Indigenous people with cancer in Australia. Both health service-based and treatment pathway-based measures were eligible. Comparators were excluded if: they did not have comprehensive assessment of patient experiences as their primary aim (including but not limited to feedback and complaint forms); their focus was patient satisfaction rather than patient experience; they largely duplicated or had the same provenance [34] as a selected source; they had been replaced by newer versions in a jurisdiction; or they were question banks [35] rather than actual tools.

The search identified two overarching categories of comparators: indicator sets used to guide measurement and patient experience surveys. Two comparators from each category were identified (Table 1).

**Table 1:** Description of selected comparators

Comparator	Provenance	Characteristics	Rationale for inclusion
**Indicator sets**			
**A** National Cancer Control Indicators Framework, Patient experience (no date) [36]	Cancer Australia. Part of National Cancer Control Indicators (NCCI) (Psychosocial care).	Eight patient experience indicators, agreed measures, questions. Derived from National Health Service (NHS) England Cancer Patient Experience Survey (CPES).	Consultative process which attempted to establish indicators for national data collection and comparison over time. Consistent with PCC Recommendation 2: ‘*A core set of nationally endorsed patient survey questions … to facilitate collation and comparison of … data in key healthcare settings’* [3] *(p2).*
**B** Prioritised cancer patient experience indicators (2017) [7]	Funded by Cancer Institute of New South Wales (NSW). Centre for Health Service Development, Australian Health Services Research Institute (AHSRI), University of Wollongong.	Prioritised list of 20 patient experience indicators. Aimed to guide service improvement.	Delphi study to identify and prioritise cancer patient experience indicators. Widely consultative work covering continuum of cancer care in general population. Ranked indicators according to their importance in cancer care. High level of consensus.
**Surveys**			
**C** Adult Admitted Patients Survey (2014, NSW) [37]	Bureau of Health Information (BHI).	Service-oriented survey: Hospital setting. Population: Adult inpatients. Purpose: Healthcare system performance, inform improvement actions, strengthen accountability [38]. Length: 106 questions from multiple sources. Mode: self-administered, mailed [39].	BHI used this standard adult inpatient survey to oversample Aboriginal people (*n* = 2682) who were NSW hospital inpatients in 2014. Differences between inpatient experiences of Aboriginal and non-Aboriginal people were reported [11]. Changes made to subsequent surveys.
**D** Victorian Cancer Patient Experience Survey (2017) [40]	Department of Health and Human Services (Victoria).	Pathway-oriented survey in modules. Population: adult cancer patients. Purpose: Quality improvement, compare care experiences, state or treatment centre level. Length: 10 modules; > 140 questions. Mode: self-administered, mailed [41].	Covers trajectory of cancer pathway. Developed as other tools were rejected; greater relevance.

*Abbreviations*: *NSW* New South Wales, *PCC Person-centred care, BHI Bureau of Health Information*

### Stage 2: mapping comparator content to topics and identifying gaps in coverage

Detailed comparison was undertaken by extracting the individual indicators or survey items from each of the comparators and mapping these to the topics from the previous research [16], which is summarised in Table 2. Each of the included comparators were examined in their entirety. Some questions in Comparators C and D (patient experience surveys) were excluded as they were not relevant (e.g. patient medical characteristics, location of treatment, or overall assessment questions). While some broad questions (e.g. ‘*Overall how satisfied were you with the treatment you received …*.? ’) may provide relevant information, they were excluded because they do not collect such information in a systematic way.

**Table 2:** Degree to which each comparator covered the topics identified by Indigenous people and health care providers

Topic: key elements^**b**^	Comparators^**a**^			
	Indicators		Surveys	
	A	B	C	D
**Feeling safe in the system**: Cultural safety; hospital surroundings in context of cultural safety; trust in system and individual staff; personal history; colonisation; identification as Indigenous; relationships; experiences of racism; Stolen Generations^**c**^; being away from one’s Country.	P	P	P	P
**Importance of Indigenous care providers**: Crucial aspect for whole pathway; access requires monitoring; lack of Indigenous care providers; benefits to staff of Indigenous care providers; importance of ACCHOs.	N	N	N	N
**Barriers to care**: Transport, accommodation, money, especially for those away from Country; requires monitoring despite programs existing; separation from family – costs of family presence.	N	P	N	P
**Role of family and friends**; Relieves fear and anxiety; accommodating family (literally and figuratively) in hospital setting; range of support needs for patient; different types of support for family; information flow; role of patient within family influences support needs.	N	P	P	P
**Effective communication and education**: Reduced ability to absorb diagnosis; check for understanding after information provision; language; relationship as facilitator of communication; listening; communication with family; unconscious bias; feeling safe to ask questions.	P	P	P	P
**Coordination of care**: Crucial and lacking; assistance to navigate and culturally safe patient navigators needed.	P	P	P	P
**Transition between services**: Lacking in coordination; culturally appropriate support services at home absent.	P	P	P	P
**Carers’ wellbeing**: Sustained periods of stress with lack of attention to carer’s needs, no follow-up.	N	P	N	P
**Palliative care**: Cultural safety of palliative care	N	N	N/A	N

^**a**^ *For more detail on comparators, see* Table 1

***A*** *Eight cancer-specific patient experience* ***indicators****, part of National Cancer Control Indicators (NCCI)* [36]*;*

***B*** *Prioritised list of 20 patient experience cancer-specific* ***indicators*** [7]*;*

***C*** *Service-oriented* ***survey****; standard adult hospital inpatient experiences of care survey* [37]*;*

***D*** *Pathway-oriented experiences of care* ***survey****; cancer-specific* [40]

*AC Adequately Captured, P Partially captured, N Not captured at all, N/A Not Applicable - not a cancer-specific comparator*

^**b**^ *As reported in Green* et al. *2018* [16]

^**c**^ *Stolen Generations; a term referring to “the generations of Indigenous children that were forcibly removed from their families by compulsion, duress or undue influence, as a result of protectionist and child welfare laws, practices and policies in place in Australia for most of the 1900s”* [42]

*Abbreviations*: *ACCHO* Aboriginal Community Controlled Health Organisation

Two researchers (MG and JC) independently conducted the mapping process for each comparator, then compared notes, discussed discrepancies, negotiated and reached consensus. The degree to which each of the baseline topics would be captured by comparator items was described using three categories:

- adequately captured (AC); comparator contained a sufficient range of indicators or questions to assess this topic;
- partially captured (P); comparator addressed some elements of the topic, but key elements were missing;
- not captured at all (N); comparator contained no reference to this topic.

Many of the comparator items could reasonably map to more than one topic; e.g. questions about the provision of information to carers or family are relevant to both ‘Effective communication and education’ and ‘Role of family and friends’. Questions were allocated to the most relevant topic and cross referenced to other topics as appropriate. Domains or areas of care as documented in the comparators helped to guide the mapping process; this included Picker PCC domains (e.g. ‘Continuity and transition’) [36], thematic areas (e.g. ‘information provision’) [7, 37], or stage in the cancer pathway (e.g. ‘Finding out what was wrong’) [40].

## Results

The degree to which key topics, as reported by Indigenous people affected by cancer and health care providers [16], are captured by the four comparators is summarised in Table 2. The mapping process used to arrive at the summary is detailed in Additional files 1 and 2:

No comparator was categorised as completely or ‘adequately’ capturing any of the key topics, and the number of topics assessed as ‘partially’ captured by the four comparators ranged from 4 to 7 (out of 9). Results for each topic are presented separately below.

### Feeling safe in the system

All four comparators were assessed as ‘partially’ capturing relevant information about whether patients felt safe in the health system, and there were common gaps across comparators. As indicated in Table 2, this topic includes aspects relating to patients’ perceptions of cultural, emotional and, to a lesser extent, physical safety. Direct exploration of patients’ experiences of racism during their care was absent across all comparators. While being treated with respect and dignity was explicit in Comparators C and D, this was considered a component of ‘communication’ in Comparator B [7] and was not mentioned in Comparator A. Cultural issues such as the emotional impact of being away from one’s Country for treatment, the perceived cultural safety of the hospital environment, and/or the use of traditional medicine, were absent from all comparators. Comparator C contained two items about culture (regarding cultural beliefs being respected by staff and dietary needs), but Comparators A, B and D did not mention culture or beliefs at all. Cultural issues may be partially addressed through assessment of the psychosocial care of patients and carers, as in Comparator B, and through questions about emotional needs and complementary therapies, as in Comparator D.

All four comparators contained items about involvement in decision making, and three (Comparators B, C and D) included items on pain management. While neither involvement in decision making nor pain management featured strongly in the qualitative interviews which informed the topics [16], these aspects of care could be considered part of ‘feeling safe in the system’. Although both survey comparators (C and D) contain an item about whether the patient identifies as Indigenous, there was no exploration about the experience of being asked this question (e.g. whether it was done in a culturally safe way).

### Importance of Indigenous care providers

None of the comparators included an item relating to access to an Indigenous care provider, though Comparator C has since added such a question [43].

### Barriers to care

Two cancer-specific comparators (B and D) explored this area in some depth, however coverage was only ‘partial’. There was no exploration of the cost of enabling family to be present in the hospital setting or about other logistical impacts of receiving treatment away from one’s Country.

### Role of family and friends

There was considerable overlap in mapping back to this topic from the three comparators (B, C and D), which contained relevant items, particularly regarding *information needs* of family and friends. Close attention was paid to this topic in the cancer-specific survey (Comparator D), however there was a gap regarding recognition of the impact of the diagnosis on family, and accommodating family in the hospital setting. This gap was also present in Comparator C, along with a lack of exploration about the support needs of family and friends.

### Effective communication and education

This topic was explored in some depth in all comparators, with emphasis on the comprehensibility and comprehensiveness of information provided, particularly in the cancer-specific comparators. The cancer-specific survey (Comparator D) also examined relationships with health staff as a facilitator of communication, however this was not as evident in the other comparators. Comparator B ranked ‘excellent communication’ as a high priority [7] and considered this to include patients’ needs and preferences, as well as treating patients with respect and dignity. There were consistent gaps regarding communication with family and the presence and impact of unconscious bias on communication. In the context of health care, implicit or unconscious assumptions about racial or ethnic groups have been found to negatively impact patient-provider interactions, treatment decisions and adherence, and health outcomes [44]; this is referred to as implicit or unconscious bias.

### Coordination of care

All comparators examined this aspect of care to some degree, ranging from whether care was organised, to exploration of experiences with a Clinical Nurse Specialist (CNS), with Comparator D the most comprehensive in its coverage. The level of detail varied, as did the level of specificity in the description of the key contact person (e.g. the person to contact if worried versus the CNS assigned to you). None of the comparators explored the perceived cultural safety of the navigator, CNS or key contact person, although Comparator D did explore the relationship between the patient and a CNS, which recognises that dysfunction in the relationship may affect experiences of care.

### Transition between services

This topic was partially addressed by all comparators, however the cultural safety of support services was not addressed.

### Carers’ wellbeing

Comparators A and C did not examine this area. Comparator D (cancer-specific) explored this area in some depth, and the psychosocial care of carers was included in Comparator B. However, the cultural safety of carer support was not assessed.

### Palliative care

This was not explicitly included in any of the cancer-specific comparators and was considered not applicable to the non-cancer-specific Comparator C. There were items in Comparators B and D which may have elicited related information, such as questions about supportive care.

## Discussion

Based on this analysis of four comparators that represent contemporary patient experience measurement activity in Australia, it is evident that there are critical gaps in the collection of information about aspects of cancer care reported as important by Indigenous people and health care providers [16]. The lack of such information is an impediment to the development of services that meet the needs of Indigenous people with cancer, their families and communities. While some key aspects of care were partially covered, such as effective communication and coordination of care, there was little to no attention given to cultural issues. In particular, there was a consistent lack of exploration about the cultural safety of care or support services. Rather than identification of gaps from a Western biomedical perspective, as is commonly reported in health discourse in Australia [26], the work reported here identifies gaps in patient experience measurement from an Indigenous perspective.

The current comparison found that experiences such as cultural safety, racism, and relationships with care providers, were inadequately covered in existing indicators and surveys. This deficiency does not reflect the centrality of culture for Indigenous people. Further, researchers have suggested that Indigenous people’s experiences of racism should be examined explicitly [21]. Within the context of ongoing colonisation, specific attention must be directed to the unique needs and experiences of Indigenous people in the Australian health system, rather than grouping them together with other populations (e.g. ‘patients experiencing social disadvantage’).

The inadequate examination of cultural issues across all comparators revealed in this examination may provide context to previous research. The reported experiences of 2682 Aboriginal patients in New South Wales (NSW) hospitals were found to be significantly different from non-Aboriginal patients regarding specific aspects of care [11]. That research used the standard adult survey form (which is Comparator C in the current study) rather than one adapted to include issues of specific importance to Aboriginal people, as the aim was to highlight differences between Aboriginal and non-Aboriginal respondents. Marked differences were found in responses regarding ‘interpersonal or relational aspects of care’ including respectfulness and whole-person care [11]. In a separate report using additional data sources, wider gaps were found in rural areas regarding communication, respect, and patient engagement [45]. Despite the differences identified, these efforts are compromised by not containing items that assess aspects of care that may be key drivers of engagement with care for Indigenous people, such as exploration of the relationship with staff as a facilitator of communication, access to Indigenous care providers, or assessing the emotional impact of being away from Country for treatment. This suggests the need for deeper enquiry to ensure that patient experience measures reflect what is important to cancer patients [7, 9].

Calls to ensure cultural safety in the health system are embedded in Australian national health policy infrastructure [20], which also advocates robust patient experience measurement processes [3, 18]. The failure to measure patient experiences from a cultural safety perspective and the impact of the setting in which cancer care is experienced, mean that superficial information may be gathered, rather than information that may be at the core of an Indigenous person’s (dis) engagement with treatment [4, 5]. As cultural safety is determined by the patient and family [18, 20, 21], the development of indicators which come from a cultural safety perspective is likely to contribute to greater relevance of patient experience measures for Indigenous people. In the current work, this particularly applied to the topics of feeling safe in health services, the importance of Indigenous care providers, barriers to care particularly when away from one’s Country, the role of family and friends, carers’ wellbeing and palliative care. Although the data which informed these topics included feedback from health professionals, these were consistent with views expressed by Indigenous people affected by cancer [16]. Several aspects of care that are usually included in patient experience measurement - e.g. timeliness (of diagnosis, or commencement of treatment); involvement in decision making; and pain management - did not feature in our earlier research [16]. However, these are recognised as important indicators of quality of care and should be included in data collection and reporting.

As has been demonstrated in the existing literature, it is insufficient to simply translate existing tools into a language or format that reaches Indigenous people [28–31]. The Picker PCC domains [33], which have informed patient experience measures in Australia for many years [39], do not originate from an Indigenous world view and may miss core principles relevant to Indigenous populations. Handley and Nembhard have suggested that ‘*additional or new permutations’* [9] (p11) of the Picker domains may be required to accurately measure person-centred care in specific populations, and they advocate research to determine core principles for what they describe as ‘*different patient types’* [9] (p11). Measurement instruments are likely to be more relevant if built ‘from the ground up’, in order to reflect an Indigenous world view and the cultural factors that impact on Indigenous health and wellbeing, such as connection to Country and cultural expression, and to take into account the context of ongoing colonisation [27]. Such activity is occurring in several areas, including the development of a Supportive Care Needs Assessment Tool for Indigenous People [28], and work to enable the measurement of cultural safety in hospitals [21]. Many of the cultural safety items put forward by Elvidge and colleagues [21] are reflected in the current study, such as access to Indigenous care providers and respect for cultural values. While unnecessary duplication of data collection is clearly undesirable, these aspects may be key to the patient experience and therefore need to remain ‘on the table’ for those involved in the design and implementation of patient experience measurement. Efforts to measure cultural safety of services and patient experience should be complementary, with the greatest impact likely to occur through close coordination of the two processes.

Clearly, patient experience measurement approaches will evolve over time, and some gaps identified in the two surveys (C and D) relate to their respective purposes and priorities [11, 41]. The ongoing examination of Indigenous patients’ care experiences [46], the inclusion of an item about access to Indigenous care providers in recent versions [47] of the service-oriented survey (Comparator C), and the recent release of a list of recommended (but as yet unvalidated) patient experience survey questions for Indigenous hospital inpatients [24], are all encouraging. Further, the authors of Comparator A acknowledge a gap in patient experience indicators for Indigenous people [48]. While mode of administration is outside the scope of this report, it should be noted that Indigenous informants have expressed a preference for face-to-face interaction with a trusted interviewer, and noted that this was likely to be crucial in understanding the experiences of Indigenous people with cancer [16]. In addition, both surveys included here are lengthy and dense, which is likely to impact adversely on participation by Indigenous people. In determining optimal approaches to collection of information about Indigenous patient experiences, it is important that the human aspect of health care is not lost in the transition to digital data collection.

### Limitations

This study did not include every measurement instrument available and was not intended to be a comprehensive review of patient experience measurement in Australia. Although efforts were made to maximise rigour, a degree of subjectivity was required to select relevant comparators, and to map detail to the topics previously identified by Indigenous people affected by cancer and health care providers. The criteria for selecting comparators were intended to aid identification of comparators that were in contemporary use and to prioritise inclusion of comparators that assessed what actually occurred during care, rather than those that assessed ‘the gap between patient expectations and experience’, i.e. satisfaction [49] (p2). The adequacy and appropriateness of response options were not considered.

## Conclusion

Respect for the cultural perspective of individuals has been identified as a key driver of patients’ engagement with health services [12, 16–18]. Failure to adequately assess experiences of care from a cultural safety viewpoint makes it difficult to identify areas for improvement in services and may compromise efforts to improve health outcomes. Existing patient experience measurement tools and the aspects of care they explore are by no means irrelevant to Indigenous people in Australia, however they do not capture many of the key aspects of Indigenous patients’ experiences of care. Addressing the gaps requires the development of strengths-based measures that reflect an Indigenous world view and that support informed decision-making by measuring the experiences of care that are most relevant to Indigenous people in Australia.

## Supplementary Information

**Additional file 1.** Key gaps identified by mapping Comparators A and B to topics reported by Indigenous people affected by cancer and health care providers.

**Additional file 2 **Key gaps identified by mapping Comparators C and D to topics reported by Indigenous people affected by cancer and health care providers, with survey questions categorised according to *‘Aspects of care’.* Further detail on this process is available on request from the corresponding author.

## Data Availability

The datasets supporting the conclusions of this article are included within the article and its additional files.
