# Supplementary material for: Measuring health care experiences that matter to Indigenous people in Australia with cancer: identifying critical gaps in existing tools
Source: Int J Equity Health. 2021 Apr 12;20:100. doi: 10.1186/s12939-021-01433-2 (PMC8042987; doi:10.1186/s12939-021-01433-2)
Supplement: Supplementary file 1 — Additional file 1. Key gaps identified by mapping Comparators A and B to topics reported by Indigenous people affected by cancer and health care providers. [file 12939_2021_1433_MOESM1_ESM.docx]

*Additional file 1. Key gaps identified by mapping Comparators A and B to topics reported by Indigenous people affected by cancer and health care providers.*

| **Topics*** | **Comparator A****  Agreed measure (Q number) | **Key gaps in Comparator A** | **Rating ***** | **Comparator B****  Rank. Indicator | **Key gaps in Comparator B** | **Rating ***** |
| --- | --- | --- | --- | --- | --- | --- |
| **Feeling safe in the system** | Proportion of patients reporting adequate involvement in decisions about care and treatment (PEx5);  Proportion of patients reporting that their views were taken into account during treatment (PEx6). | Cultural safety regarding surroundings + personal.  Experience of racism.  Trust in staff or system.  Expressions of culture e.g. traditional medicine, emotional impacts of being away from Country.  Questions about culture or beliefs.  Experience of being asked about Indigenous status. | P | 9. Needs assessment  10. Provision of Psychosocial care (patients)  11. Shared decision making  16. Doctor’s knowledge and expertise.  17. Safety (pain)  19. Safety (medication) | Cultural safety regarding surroundings + personal.  Experience of racism.  Trust in staff or system.  Expressions of culture e.g. traditional medicine, emotional impacts of being away from Country.  No questions about culture or beliefs.  Experience of being asked about Indigenous status. | P |
| **Importance of Indigenous care providers** | None | Access to ALO/AHW (+point of care).  Access to ACCHO. | N | None | Access to ALO/AHW (+point of care).  Access to ACCHO. | N |
| **Barriers to care** | None | Logistical barriers to care: travel, accommodation, finance.  Logistical impacts of being away from Country.  Costs of family presence at hospital. | N | 2. Access to care (all modalities)  13. Access (location)  15. Access (logistics) | Logistical impacts of being away from Country.  Costs of family presence at hospital. | P |
| **Role of family and friends** | None | Accommodating family in hospital.  Support needs of family.  Impact of diagnosis on family. | N | (Overlap with Effective communication and education) | Accommodating family in hospital.  Support needs of family.  Impact of diagnosis on family. | P |
| **Effective communication and education** | Proportion of patients: - reporting they understood their diagnosis (PEx1);  - who were given written information about diagnosis (PEx2);  - reporting that the possible side effects of treatments were explained in an understandable way (PEx7);  - given written information about the side effects of treatments (PEx8). | Exploration of relationship as facilitator of communication, e.g. listened to.  Who to communicate with in family.  Unconscious bias.  Encouragement of questions. | P | 4. Communication (excellent patient-centred communication, including respect, dignity, needs and preferences)  6. Information provision (side effects, carers included)  8. Information style (comprehensibility)  11. Information provision (tailored and accurate) | Unconscious bias.  Encouragement of questions. | P |
| **Coordination of care** | Proportion of patients:  - offered a written assessment and care plan (PEx3);  - given the name of a CNS for treatment support (PEx4). | Cultural safety of navigator. | P | 1. Coordinated care  3. Timeliness of first treatment  6. Timeliness of diagnosis  17. Care plan | Cultural safety of navigator. | P |
| **Transition between services** | As for Coordination of care | Coordination of transition between services.  Cultural safety of support services. | P | 5. Follow-up care / survivorship | Cultural safety of support services. | P |
| **Carers’ wellbeing***.* | None | Carer’s wellbeing and follow-up needs.  Assessment of cultural safety of support provided to carer. | N | 10. Psychosocial care (carers) | Assessment of cultural safety of support provided to carer. | P |
| **Palliative care** | None | Cultural safety of palliative care. | N | Not specified, but supportive care assessed. | Cultural safety of palliative care. | P |

** As reported in Green et al. 2018 (16). For detailed elements of topics, see Table 2.*

*** Comparators:*

***A*** *Eight cancer-specific patient experience indicators, part of National Cancer Control Indicators (NCCI) (36);*

***B*** *Prioritised list of 20 patient experience cancer-specific indicators (7).*

*For more detail, see Table 1.*

**** Degree to which each comparator covered the topics identified by Indigenous people and health care providers.*

*AC; Adequately Captured:*

*P; Partially captured:*

*N; Not captured at all:*

*N/A; Not Applicable - not a cancer-specific comparator.*

*Abbreviations: ALO; Aboriginal Liaison Officer: AHW; Aboriginal Health Worker: ACCHO; Aboriginal Community Controlled Health Organisation.*
