# Supplementary material for: Measuring health care experiences that matter to Indigenous people in Australia with cancer: identifying critical gaps in existing tools
Source: Int J Equity Health. 2021 Apr 12;20:100. doi: 10.1186/s12939-021-01433-2 (PMC8042987; doi:10.1186/s12939-021-01433-2)
Supplement: Supplementary file 2 — Additional file 2 Key gaps identified by mapping Comparators C and D to topics reported by Indigenous people affected by cancer and health care providers, with survey questions categorised according to ‘Aspects of care’. Further detail on this process is available on request from the corresponding author. [file 12939_2021_1433_MOESM2_ESM.docx]

*Additional file 2. Key gaps identified by mapping Comparators C and D to topics reported by Indigenous people affected by cancer and health care providers (using ‘**Aspects of care’).*

| **Topic*** | **Comparator C****  Indicators measured | **Key gaps in Comparator C**  Questions about: | **Rating ***** | **Comparator D****  Indicators measured | **Key gaps in Comparator D**  Questions about: | **Rating ***** |
| --- | --- | --- | --- | --- | --- | --- |
| **Feeling safe in the system** | Cultural/religious beliefs respected.  Dietary needs related to culture.  Treated with respect, courtesy, kindness.  Privacy.  Trust in staff categories (Doctor/Nurse).  Able to talk with staff if needed.  Involvement in decision making.  Timeliness: of care: waiting times.  Pain management  Feelings of safety (discuss worries, call bell, assistance if needed, complaints, ID check).  Identification as Indigenous. | Cultural safety regarding surroundings + personal.  Experience of racism.  Trust in system.  Expressions of culture e.g. traditional medicine, emotional impact of being away from Country.  Emotional needs (directly).  Experience of being asked about Indigenous status. | P | Treated with respect, courtesy, kindness.  Emotional needs.  Able to talk with staff if needed.  Involvement in decision making.  Timeliness (of care, waiting times).Pain management  Feeling of safety (discuss worries, assistance if needed, complaints outlet).  Complementary therapies.  Identification as Indigenous. | Cultural safety regarding surroundings + personal.  Experience of racism.  Trust in staff or system.  Expressions of culture e.g. traditional medicine, emotional impacts of being away from Country.  Privacy.  Culture or beliefs.  Experience of being asked about Indigenous status. | P |
| **Importance of Indigenous care providers** | None, but later version does include question about access to ALO. | Access to ALO/AHW (+ which point of care).  Access to ACCHO. | N | None | Access to ALO/AHW, (+ which point of care).  Access to ACCHO. | N |
| **Barriers to care** | Not measured. | Logistical barriers to care: travel, accommodation, finance.  Logistical impacts of being away from Country.  Costs of family presence at hospital. | N | Many questions about finance, accommodation and travel needs. | Logistical impacts of being away from Country.  Costs of family presence at hospital. | P |
| **Role of family and friends** | Family and friends: Information needs.  Family: access to doctor. | Accommodating family in hospital.  Support needs of family.  Impact of diagnosis on family. | P | Family and friends: information and support needs. | Accommodating family in hospital.  Impact of diagnosis on family. | P |
| **Effective communication and education** | Information provision: comprehensiveness; quantity; comprehensibility.  Need for interpreter. | Exploration of relationship as facilitator of communication, e.g. listened to.  Who to communicate with in family.  Unconscious bias.  Encouragement of questions. | P | CNS and CNS relationship as facilitator of communication.  Information provision: comprehensiveness; quantity; comprehensibility; check understanding; knowledge of various health services.  Need for interpreter.  Encouragement of questions. | Who to communicate with in family.  Unconscious bias. | P |
| **Coordination of care** | Knowledge of staff re patient history.  Staff working together. | Navigator/ CNS – key contact person.  Whether navigator culturally safe. | P | Many questions; coordination, information sharing, staff working together, conflicting advice. | Whether CNS (*navigator*) culturally safe (but does explore CNS relationship). | P |
| **Transition between services** | Transition: readiness for discharge; home situation, information/support needs, who to contact, medication, discharge day. | Cultural safety of support services. | P | Transition: information and support needs, home situation, coordination of care, who to contact. | Cultural safety of support services. | P |
| **Carers’ wellbeing** | Not measured. | Carers’ wellbeing, follow-up needs.  Assessment of cultural safety of support provided to carer. | N | Family / friends: Support needs of family/friends.  Whether patient knew how to get support for family. | Assessment of cultural safety of support provided to carer. | P |
| **Palliative care** | (Not cancer specific) | (Not cancer specific) | N/A | Not mentioned, though supportive care assessed. | Cultural safety of supportive care | N |

*Notes: To present this data clearly, survey questions were categorised into ‘Aspects of care’. Further detail on this process is available from the corresponding author.*

** As reported in Green et al. 2018 (16). For detailed elements of topics, see Table 2.*

*** Comparators:*

***C*** *Service-oriented survey; standard adult hospital inpatient experiences of care survey (37);*

***D*** *Pathway-oriented experiences of care survey; cancer-specific (38).*

*For more detail, see Table 1.*

**** Degree to which each comparator covered the topics identified by Indigenous people and health care providers:*

*AC, Adequately Captured;*

*P, Partially captured;*

*N, Not captured at all;*

*N/A, Not Applicable - not a cancer-specific comparator.*

*Abbreviations: ALO; Aboriginal Liaison Officer: AHW; Aboriginal Health Worker: ACCHO; Aboriginal Community Controlled Health Organisation: CNS; Clinical Nurse Specialist.*
